# Characterizing Universal Object Representations Across Vision Models

**Published:** 2026-05-13

**Authors:** Florian P. Mahner, Johannes Roth, Ka Chun Lam, Michael F. Bonner, Francisco Pereira, Martin N. Hebart

**Affiliations:** Vision and Computational Cognition Group, Max Planck Institute, Justus-Liebig-University Giessen; Vision and Computational Cognition Group, Max Planck Institute, Justus-Liebig-University Giessen; Machine Learning Core, National Institute of Mental Health, Bethesda, MD, USA; Department of Cognitive Science, Johns Hopkins University, Baltimore, MD, USA; Machine Learning Core, National Institute of Mental Health, Bethesda, MD, USA; Vision and Computational Cognition Group, Max Planck Institute, Justus-Liebig-University Giessen

## Abstract

Deep neural networks trained with different architectures, objectives, and datasets have been reported to converge on similar visual representations. However, what remains unknown is which visual properties models actually converge on and which factors may underlie this convergence. To address this, we decompose the object similarity structure of 162 diverse vision models into a small set of non-negative dimensions. To determine universal versus model-specific dimensions, we then estimate how often each dimension reappears across models. In contrast to model-specific dimensions, universal dimensions are more interpretable and more strongly driven by conceptual image properties, indicating the relevance of interpretability and semantic content as implicit factors driving universality across models. Differences in architecture, objective function, training data, model size, and model performance do not explain the emergence of universal dimensions. However, models with more universal dimensions also better predict macaque IT activity and human similarity judgments, suggesting that universality reflects representations relevant to biological vision. These findings have important implications for understanding the emergent representations underlying deep neural network models and their alignment with biological vision.

## 1 Introduction

Deep neural networks have become central to visual representation learning [1] and are increasingly used as computational models of the visual system [2–4]. Recent evidence suggests that deep neural networks trained on visual tasks converge on a common representational structure, despite substantial differences in architecture, objective function, and training data [5, 6]. This shared structure aligns more closely with neural responses in human visual cortex than model-specific structure [7, 8], suggesting that these recurring representations may matter for biological vision, as well.

However, what properties these shared representations capture and what factors determine their emergence remain poorly understood. First, it is unclear whether universal representations reflect high-level conceptual properties such as object categories, or perceptual image properties such as edges and colors that have long been known to be shared across models [6, 9]. Second, prior work has shown that training data and objective often matter more than architecture for alignment with brains and behavior [10, 11], but which of these factors drive universality across a large and diverse set of vision models, and how universal properties relate to biological vision, has remained open.

Addressing these questions requires us to overcome several challenges. First, we need to make representations across a wide range of different model architectures directly comparable. Second, we need to find dimensions underlying these representations that decompose an image representation at a level of granularity suitable for cross-model comparisons, since overly fine-grained representations (e.g. individual neurons) lack a canonical correspondence across models. We address these questions by decomposing the internal representations of 162 diverse vision models [11] for 22,248 object images from the THINGS database [12] into a small set of non-negative dimensions, following cognitive-science work showing that human similarity judgments for natural objects are captured by a similarly small, interpretable, non-negative representation [13]. We then quantify the universality of each dimension by measuring how consistently it recurs across models. Together, the loadings and universality score make it possible to characterize what properties make up universal and model-specific dimensions, which inductive biases are related to universality, and how universality aligns with biological vision. Our contributions are as follows:

- We find that vision models, despite their differences, share a set of interpretable universal dimensions.
- We demonstrate that universal dimensions are more interpretable and more strongly explained by conceptual image properties than model-specific dimensions, which are less interpretable and more strongly driven by perceptual image properties.
- By isolating the role of architecture, objective function, and training data, we find that no single inductive bias accounts for universality, nor does model size or downstream performance.
- Instead, we show that universal dimensions are highly predictive of multi-unit activity in macaque inferior temporal cortex and of human behavioral similarity judgments, suggesting that universal dimensions share constraints with biological vision.

## 2 Related Work

Our work relates to two broad research lines, one on representational alignment, and one on concept-based interpretability. Work on representational alignment has shown that vision models can differ substantially in what they learn: They rely more on texture than on shape relative to humans [14, 15], produce distinct intermediate representations across random seeds [16], and differ in overall representational geometry [17]. The Platonic Representation Hypothesis proposes that, despite differences in architecture, objective function, and training data, model representations become increasingly similar with scale and even across modalities [5], though recent work has questioned this and suggested a more nuanced picture [18–20]. Other work has examined a related question within vision, asking whether diverse models share universal structure and whether these shared components are especially relevant for neural alignment [6–8]. Across work relating models to neural responses and behavior, training data and objective function often matter more than architecture or scale once other factors are controlled [10, 11, 21–24]. These studies, however, mainly establish *whether* models overlap, not *what properties* the shared representations actually capture. Concept-based interpretability instead asks what models have learned in human-understandable terms [25, 26], rather than merely visualizing the most predictive image regions [27]. A useful distinction has been made between concept discovery, which extracts interpretable units from a representation, and concept importance estimation, which measures their contribution to model outputs [28]. Early methods tested whether models represented fixed human-labeled concepts [25, 26], while later work recovered concepts directly from activations [29–35]. More recent work used sparse autoencoders to describe a model by a large set of directions in its activation space, where each direction corresponds to a concept the model has learned [36–40], and Thasarathan et al. [41] extended this to multiple models by jointly training a single sparse autoencoder across a small set of pretrained models to recover a shared set of directions. These approaches operate in each model’s activation space, so comparing many models requires aligning spaces and matching across thousands of features. We therefore describe each model by the similarity relationships its activations induce over a fixed set of objects, and decompose these similarities into a small number of non-negative dimensions defined as loadings over the same stimuli. Most closely related to our work, Hebart et al. [13] showed that human similarity judgments can be explained by a sparse, non-negative embedding whose dimensions are reproducible, interpretable, and predictive of behavior. Mahner et al. [9] derived sparse, non-negative embeddings for a small set of deep neural networks and found that their dimensions were dominated by visual rather than semantic properties. We extend this line of work to a large sample of vision models and introduce a measure of how consistently each dimension recurs across them.

## 3 Methods

### 3.1 Notation

Let $𝒳=\left\{x_{1},\ldots ,x_{N}\right\}$ be a dataset of N object images, and let $ℱ=\left\{f_{1},\ldots ,f_{M}\right\}$ be a collection of M vision models. For an image $x_{i}\in 𝒳$, each model $f_{m}\in ℱ$ extracts a $d_{m}$-dimensional feature vector from its penultimate layer, denoted as $z_{m}\left(x_{i}\right)\in R^{d_{m}}$. Note that the dimensionality $d_{m}$ may vary across models. We used the penultimate layer because it is closest to the behavioral output and serves as the final stage of representation, integrating high-level conceptual and fine-grained perceptual information to produce an output. For each model $f_{m}$, we collected the representations of all images in 𝒳 into a matrix $Z_{m}\in R^{N\times d_{m}}$, where the i-th row is given by $z_{m}{\left(x_{i}\right)}^{⊤}$.

### 3.2 Dataset and Models

We used the THINGS image database [12], which spans 1,854 basic-level object categories. For each object category, we selected 12 image exemplars, yielding a dataset 𝒳 with a total of N=22,248 images. We chose THINGS since it was designed to span a broad range of object concepts, is not part of common training sets, and has associated neural and behavioral data, making it well suited for evaluating learned representations against biological vision. Using THINGS, we extracted penultimate-layer representations from M=162 vision models applied to the images in 𝒳. Our model set ℱ comprised those in Conwell et al. [11] (excluding IPCL models), plus six OpenCLIP ViT-L/14 models [42]. The resulting set spans four architecture classes (99 convolutional, 51 transformer, 9 MLP-Mixer, and 3 hybrid), a range of objective functions (supervised classification, self-supervised learning, vision-language contrastive learning, and one untrained baseline), and diverse training datasets (including ImageNet, ImageNet-21k, YFCC15M, LAION, and Taskonomy). The full list of models is provided in Table S1. This diversity enables controlled comparisons that disentangle the effects of architecture, objective, and training data on our universality metric.

### 3.3 Symmetric Nonnegative Factorization

**Figure 1:**
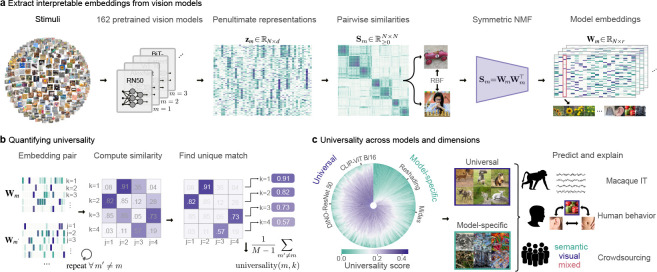
Overview of the analysis pipeline and universality framework. **(a)** For each of 162 vision models, we extract penultimate-layer representations for object images, compute pairwise object similarity matrices, and apply symmetric nonnegative matrix factorization to obtain non-negative embeddings. **(b)** We compute a universality score that quantifies how consistently each dimension of a model’s embedding reappears across all other models. **(c)** Left: distribution of per-dimension universality scores across all 162 models and 50 dimensions per model. Right: universal and model-specific dimensions are used to predict neural activity in macaque IT cortex and human similarity judgments, and to label the dimension content via online crowdsourcing.

To make dimensions comparable across models with different feature bases and interpretable in terms of the images that define them, we characterize each model by the similarity structure it induces over a common image set and decompose that structure into a small set of nonnegative dimensions (Fig. 1a).

#### Generate representational similarity matrix

Models in ℱ produce features in spaces of different dimensionalities and different bases, and several approaches exist for comparing such representations [17, 43]. We work at the level of similarity structure [44], characterizing each model by the pairwise similarities it induces over the images in 𝒳. This level of description is invariant to rotations of each model’s feature basis and yields a common N×N matrix for every model in ℱ. Because similarity is determined by the pairwise distances between image representations, feature dimensions that vary little across 𝒳 contribute little to these distances, so the similarity matrix emphasizes the structure along which the images actually differ. Similarity is defined over the shared image set, so any dimensions we extract from these matrices are automatically indexed by the same images across models and can be compared directly. For each model $f_{m}$, we computed a symmetric, entrywise nonnegative similarity matrix $S_{m}\in R_{\geq 0}^{N\times N}$ via a radial-basis function (RBF) kernel:

(1) $${\left[S_{m}\right]}_{i,j}=exp\left(-\frac{{\left\|z_{m}\left(x_{i}\right)\;-\;z_{m}\left(x_{j}\right)\right\|}^{2}}{2\sigma_{m}^{2}}\right),$$

where $\sigma_{m}$ is chosen per model to jointly maximize factorization stability and the explained variance of the low-rank reconstruction. Concretely, we searched over multipliers α of the median pairwise Euclidean distance ${\tilde{d}}_{m}$ [45], setting $\sigma_{m}=\alpha^{*}\cdot {\tilde{d}}_{m}$, where $\alpha^{*}$ maximizes the harmonic mean of both criteria (Appendix B.1). We used the RBF kernel because it is a common choice for similarity measures [17], and it maps similarities to the interval [0, 1] and thereby supports interpretability for the subsequent nonnegative factorization step. Furthermore, the RBF kernel guarantees that $S_{m}$ is positive semi-definite and nonnegative, both of which are required properties for symmetric nonnegative factorization (Eq. 2).

#### Decompose similarity matrix into interpretable image embeddings

We decomposed each similarity matrix into r non-negative dimensions using symmetric NMF [46]. For a chosen rank r, we estimated a nonnegative embedding $W_{m}\in R_{\geq 0}^{N\times r}$ of all images in 𝒳 by solving:

(2) $$\underset{W_{m}\geq 0}{min}\frac{1}{2}{\left\|S_{m}-W_{m}W_{m}^{⊤}\right\|}_{F}^{2}.$$

Each image is represented by a row of the matrix $W_{m}$. Each column $w_{m,k}\in R_{\geq 0}^{N}$ defines a *dimension*: its entries provide a nonnegative loading for each object image, and the numeric weight shows how strongly that image contributes to dimension k. The nonnegativity constraint promotes an interpretable parts-based factorization [46]. We optimized Eq. 2 using block-successive upper-bound minimization [47], following the authors’ extension (Appendix B.2), independently for each model and for ranks r∈{10,30,50,100,200}. For each model, rank, and candidate bandwidth multiplier, we ran B=5 random initializations. The bandwidth was selected by the harmonic mean of factorization stability and explained variance (Appendix B.1); within the selected bandwidth, we retained the most central seed (Appendix B.2). For all model comparisons, we fixed rank to r=50. In Appendix B.3, we show that our main results are insensitive to this choice.

### 3.4 Universality

We quantified how consistently a dimension recurs across models (Fig. 1b). Given a dimension k in model $f_{m}$ and a dimension j in model $f_{m^{\prime }}$, we measured their agreement via squared cosine similarity, which is scale-invariant, bounded, and consistent with the Frobenius-norm objective of symmetric NMF:

(3) $$s\left(w_{m,k},w_{m^{\prime },j}\right)={cos}^{2}\left(w_{m,k},w_{m^{\prime },j}\right)=\frac{{\left(w_{m,k}^{⊤}w_{m^{\prime },j}\right)}^{2}}{{\left\|w_{m,k}\right\|}^{2}{\left\|w_{m^{\prime },j}\right\|}^{2}}\in [0,\;1].$$

Because symmetric NMF factors are identifiable only up to permutation, we established correspondences between models through a one-to-one assignment. Unlike a greedy best-match, which can allow multiple target dimensions to map onto the same source dimension, this formulation enforces a bijection across the full set of dimensions (Appendix C.1). For each model pair ($m,m^{\prime }$), we determined the optimal permutation $\pi_{m,m^{\prime }}^{*}\in {\arg max}_{\pi \in {𝒫}_{r}}\sum_{k=1}^{*}s\left(w_{m,k},w_{m^{\prime },\pi (k)}\right)$ via the Hungarian algorithm and defined the per-dimension universality as:

(4) $$\text{universality}\;(m,k)=\frac{1}{M\;-\;1}\sum_{m^{\prime }\neq m}s\left(w_{m,k},w_{m^{\prime },\pi^{*}(k)}\right)\text{.}$$

As all dimensions are nonnegative, any two of them will share some positive cosine similarity even if unrelated, and denser dimensions will have a higher baseline than sparser ones. This could distort the ranking of universality across dimensions. We corrected for this by calibrating against a permutation null that shuffles the row order of $W_{m^{\prime }}$, destroying stimulus correspondence while preserving column structure (Appendix C.1). After calibration, values near 1 indicate dimensions that are consistently recovered across models, while values near 0 indicate dimensions with chance-level cross-model agreement.

## 4 Results

### 4.1 Universal dimensions are shared across vision models

We first computed universality scores across 8,100 dimensions from 162 models. Scores ranged from near chance (0.0003) to 0.54 (Fig. 1c), with the upper end reaching 64% of the within-model stability ceiling, estimated from independent runs of the same model (median ceiling 0.84, Appendix C.2). Most models contained both highly universal and model-specific dimensions, with 80% having dimensions in both the top and bottom quartiles. To confirm that these findings are not driven by the specific choice of images or models, we recomputed universality scores after (i) changing the image set, (ii) bootstrap-resampling the model set, and (iii) excluding entire model families. As an alternative image set, we used ObjectNet [48], which depicts objects in cluttered real-world scenes with varied viewpoints and rotations, in contrast to the object-centered images in THINGS. Universality scores recomputed from ObjectNet embeddings largely preserved the model ranking (ρ=0.81). Bootstrap resampling to 20% of models (n=32) yielded a highly consistent ranking (ρ=0.97; Fig. S6a), and excluding model families also left the ranking largely intact (ρ=0.83; Fig. S6b). These controls confirm that universality is a stable property of how models organize object representations (see Appendix C.3 for details).

### 4.2 Universal dimensions are biased towards interpretable, conceptual image properties

**Figure 2:**
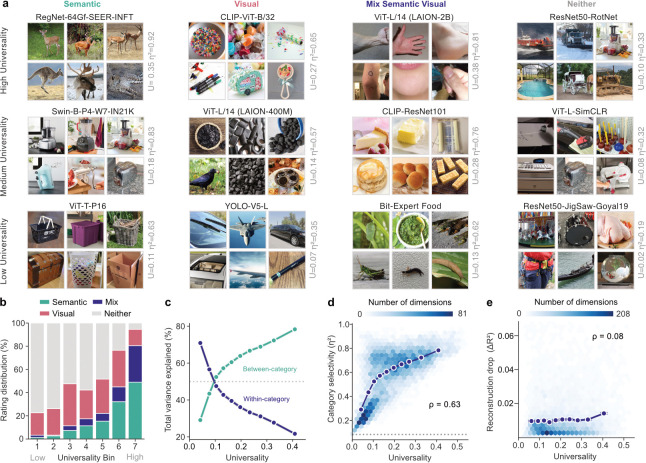
Universal dimensions are more driven by conceptual object properties, and model-specific dimensions by visual properties or lack interpretable structure. **(a)** Representative model dimensions for each label category (columns) at low, mid, and high universality (rows). Each grid shows the top-weighted THINGS images for that dimension. **(b)** Proportion of dimensions assigned each human label (semantic, visual, mix semantic-visual, neither) across all universality bins. At low universality, 78% of dimensions are rated as uninterpretable (*neither*); at high universality, semantic and mixed dimensions together account for 80%. **(c)** Between- and within-category image loading variance across universality deciles, pooled over all 162 models. The most universal dimensions (top decile) are dominated by between-category variance (~78%), while the most model-specific dimensions (bottom decile) are dominated by within-category variance (~71%). **(d)** Category consistency ($\eta^{2}$) vs. universality for all dimensions (ρ=0.63). **(e)** Reconstruction importance ($\Delta R^{2}$) vs. universality. The near-zero pooled correlation (ρ=0.01 across all dimensions; within-model median ρ=0.08) indicates that model-specific dimensions contribute as much to a model’s similarity structure as universal ones.

We next asked what image properties distinguish universal from model-specific dimensions (Fig. 2a). Some dimensions have high values for images sharing high-level object concepts such as animals, food, or household objects, while others group images by lower-level perceptual properties such as color or texture, and some show no clear pattern at all. To quantify the relationship between the dimension content and its universality, we asked crowd-sourced participants to label 1,059 representative dimensions, selected via clustering from the full set of 8,100, as *semantic*, *visual*, *both*, or *neither* (see Appendix H for details). Ratings were reliable, with median per-rater agreement with the majority label of 0.67 and median split-half agreement of 0.57. Binning the dimensions by their universality score revealed a sharp change in interpretability (Fig. 2b). At low universality, 78% of dimensions were labeled *neither*, indicating that model-specific dimensions largely lack interpretable structure. At high universality this fraction collapsed to 4%, indicating that universal dimensions were more interpretable to raters. Among the dimensions deemed interpretable, the content also varied with universality. The *semantic* and *both* labels together accounted for 80% of dimensions at high universality, while the *visual* label remained flat across the universality range. Thus, universal dimensions capture high-level object concepts, whereas the interpretable model-specific dimensions reflect low-level visual features.

Moreover, if universal dimensions capture conceptual object structure, we would expect them to show stronger organization around basic object categories than model-specific ones. Because THINGS provides 12 exemplars for each of 1,854 categories, we can decompose each dimension’s loading variance into between- and within-category components. High between-category variance indicates that the dimension groups images by object category, while high within-category variance indicates that it varies independently of category. We formalize this as category consistency $\eta^{2}$, the fraction of variance explained by category membership (see Appendix D):

(5) $$\eta^{2}=\frac{{SS}_{\text{between}}}{{SS}_{\text{total}}}.$$

A high $\eta^{2}$ indicates grouping by object category, whereas a low $\eta^{2}$ reflects within-category variation in visual features such as texture, viewpoint, or color. Category consistency ($\eta^{2}$) correlates strongly with universality across all 8,100 dimensions (ρ=.63; Fig. 2d). To see how this relationship changes across the universality spectrum, we binned all dimensions into deciles and computed the fraction of variance that falls between versus within categories in each bin (Fig. 2c). The most universal dimensions (top decile) are dominated by between-category variance (~78%), while for the most model-specific dimensions (bottom decile), within-category variance dominates (~71%). This confirms that category-level object structure, a key component of the semantic labels assigned by human raters, is strongly associated with universality.

Universal dimensions thus capture conceptual object structure and are more interpretable, but does this mean they play a larger role in a model’s similarity structure? We computed each dimension’s reconstruction importance, the drop in variance explained in the similarity matrix when that dimension is removed. Universality and reconstruction importance show near-zero correlation (within-model median ρ=0.08; Fig. 2e), indicating that universality reflects conceptual content but not contribution to similarity structure.

### 4.3 Model inductive biases do not determine universality

If universality is a general property of learned visual representations, it should emerge across different architectures, objective functions, and training datasets rather than only under a specific design choice. To assess this, we first computed a single universality score per model, averaging across its 50 dimensions. This makes it possible to compare models that differ in one design choice while holding the others approximately constant. Model-level universality showed no reliable correlation with ImageNet top-1 accuracy (ρ=-0.04, p=0.72; n=101) or number of parameters (r=0.08, p=0.31; n=162), ruling out model performance or size as trivial explanations (Fig. S5). We tested each of the five pre-specified controlled contrasts with a single uniform procedure. Two-group contrasts were tested with Welch’s t, multi-group contrasts with one-way ANOVA, and contrasts whose groups were all singleton were reported descriptively, all with Bonferroni multiple comparison correction. We report Hedges’ g for two-group contrasts and $\omega^{2}$ for multi-group contrasts with 95% confidence intervals.

#### Architecture.

**Figure 3:**
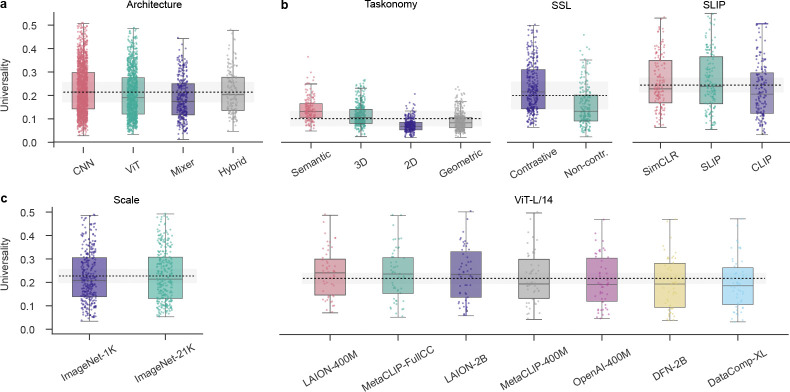
Controlled comparisons of per-dimension universality. Each panel varies one factor while holding the others approximately constant. Box plots show the distribution of per-dimension universality scores; individual dots are dimensions, colored by model. The dashed line and gray band indicate the grand mean ± 1 SD across all models in that group. **(a)** Architecture: 34 CNNs, 21 transformers, 6 MLP-Mixers, and 3 hybrids, all trained on ImageNet-1K classification. **(b)** Objective function: Taskonomy ResNet-50 encoders grouped by task cluster (n=23, excluding two singleton clusters); contrastive vs. non-contrastive self-supervised ResNet-50 models (n=10); SLIP ViT models trained with SimCLR, SLIP, or CLIP (n=9). **(c)** Training data. Left: ImageNet-1K vs. ImageNet-21K, matched by architecture (n=12). Right: seven ViT-L/14 CLIP models trained on different web-scale datasets.

We compared 34 CNNs, 21 transformers, 6 MLP-Mixers, and 3 hybrids, all trained on ImageNet-1K classification (n=64). Architecture had no statistically significant effect on universality (F(3,60)=2.54, p=0.33, $\omega^{2}=0.07$ with 95% CI [−0.02, 0.29]; Fig. 3a), indicating that universality is not tied to a specific architecture class.

#### Objective function.

We analyzed 25 ResNet-50 encoders from the Taskonomy benchmark [49], each trained on a different visual task with identical architecture and training data. These models showed low universality overall (cluster means ranging from 0.05 to 0.12, well below the full-set median of 0.22), reflecting the narrow distributions of their training tasks. Nevertheless, within this group, task cluster had a clear effect on the relative level of universality after excluding two singleton clusters (F(3,19)=12.7, p<.001, $\omega^{2}=0.60$; Fig. 3b, left). Semantic tasks (object/scene classification) produced the most universal dimensions, followed by 3D, Geometric, and 2D tasks. Across individual tasks, universality spanned a range of 0.125, from denoising at the bottom to object classification at the top. We next examined whether the choice of self-supervised learning objective affects universality. Among 10 self-supervised ResNet-50 models trained on ImageNet, contrastive objectives (SimCLR, MoCo, Barlow Twins, SwAV, DeepCluster) showed nominally higher universality than non-contrastive methods (RotNet, Jigsaw, ClusterFit), but the effect was not significant after correction (Welch t=3.21, p=.065, Hedges’ g=1.76; Fig. 3b, center). Finally, to isolate the effect of language alignment, we compared ViT models from the SLIP family [50], all trained on YFCC15M but with different objectives, SimCLR (purely visual), CLIP (vision-language), and SLIP (combined) (n=9). Although CLIP models scored lower in universality than SimCLR and SLIP, the difference was not statistically significant (F(2,6)=3.80, p=.43, $\omega^{2}=0.38$; Fig. 3b, right).

#### Training data.

We compared six models trained on ImageNet-1K with six trained on ImageNet-21K, matched by architecture family [11]. Data scale had no effect on universality (Welch t=-0.33, p>.99, Hedges’ g=-0.18; Fig. 3c, left). To further isolate the role of training data, we compared seven ViT-L/14 CLIP models that share the same architecture and objective but differ in training dataset (OpenAI WIT, LAION-400M, LAION-2B, DataComp-XL, DFN-2B, MetaCLIP-400M, MetaCLIP-FullCC). Universality scores within this group varied significantly less than expected from a random sample of seven models (bootstrap test on within-group standard deviation with null drawn from all 162 models, one-sided p=.009,10,000 resamples; Fig. 3c, right). Together, neither dataset scale nor training-data choice substantially changed universality. Overall, although training objective may slightly modulate universality in specific model subsets, no single inductive bias accounts for universality. Universality thus emerges across diverse design choices, suggesting that it reflects a general property of learned visual representations.

### 4.4 Universality predicts neural and human behavioral alignment

So far, the results have established that universal dimensions capture high-level conceptual object properties and that they emerge across diverse architectures, objective function, and training datasets rather than being driven by any single design choice. This leaves open why different models converge on these particular dimensions. A plausible explanation is that universal dimensions reflect aspects of the visual world itself rather than consequences of any particular model design. If so, universal dimensions should align with biological visual representations, since both are shaped by the same visual world, whereas model-specific dimensions reflect idiosyncrasies of particular models. We tested this using neural recordings from macaque inferior temporal (IT) cortex and human behavioral judgments, both collected on the THINGS image set.

#### Prediction of macaque IT neural recordings

**Figure 4:**
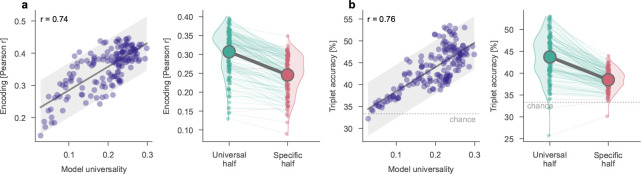
Universality predicts neural and behavioral alignment. **(a)** Left: model universality vs IT encoding accuracy (mean across two macaques); Right IT encoding accuracy from the universal vs specific half of each model’s dimensions. **(b)** Left: model universality vs human triplet accuracy; Right: triplet accuracy from the universal vs specific half. Dotted lines are chance level (1/3).

To quantify the alignment between model dimensions and neural activity, we predicted multi-unit activity in macaque IT cortex from each of the 162 models’ dimensions using cross-validated ridge regression (see Appendix E for details). Models with more universal dimensions also predict IT activity more accurately (r=0.74, p<.001; Fig. 4a). To test whether this is driven by universal dimensions specifically, we split each model’s dimensions at the median universality score into a universal and a model-specific half. The universal half predicts IT activity significantly better than the model-specific half (paired *t*-test, p<.001).

#### Prediction of human behavior

To quantify the alignment between model dimensions and behavior, we evaluated the 162 models against a dataset of odd-one-out judgments [13], in which participants viewed triplets of object images and chose the image least similar to the other two. For each model, we predicted the human choice from pairwise cosine similarities between the embeddings $W_{m}$ of the three images (see Appendix F for details). Models with more universal dimensions also predict human judgments more accurately (r=0.76, p<.001; Fig. 4b), and the universal half of each model’s dimensions yields significantly higher accuracy than the model-specific half (0.42 vs. 0.37, chance = 0.33, paired *t*-test, p<.001).

## 5 Discussion and Conclusions

We decomposed the representations of 162 vision models into interpretable dimensions using symmetric NMF and quantified how consistently each dimension reappears across models. Across this diverse set of models, we found that many dimensions appear repeatedly. These universal dimensions are also the ones that are most interpretable to human observers and most strongly tied to conceptual, between-category structure. In contrast, model-specific dimensions display higher within-category variation, are less interpretable, and relate more to lower-level visual properties.

Our controlled comparisons show that no single inductive bias – architecture, objective function, or training data – accounts for universality. Taskonomy models show uniformly low universality relative to the broader model zoo, though, within this family, semantic tasks produce more universal dimensions than geometric or 2D tasks. Instead, universality is more closely aligned with biological vision than with any inductive bias. Universal dimensions predict both neural activity in macaque IT cortex and human behavioral similarity judgments more accurately than model-specific ones. The very same dimensions that align with brains and behavior are also the ones that human raters find interpretable and label as semantic. Interpretability, conceptual content, and biological alignment thus co-occur in the structure that diverse models converge on. This suggests that universality is an emergent property of successful visual representation learning.

A growing body of work has provided evidence for convergence and shared structure across diverse models [5, 7, 8, 18–20]. Our results are consistent with the view that there may be universal dimensions of visual representation, with diverse models converging on conceptual object properties. However, this convergence is far from complete. The most universal dimensions do not have a perfect universality score, and model-specific dimensions, which capture lower-level visual properties, contribute equally to each model’s representational geometry.

This work addresses a basic question about visual representation learning: what structure diverse models converge on when they learn to represent objects. We find that this structure is conceptual, interpretable, and aligned with biological vision. Understanding why it emerges so consistently across very different architectures, objectives, and training regimes may help clarify the underlying principles shared by artificial and biological vision. More broadly, our approach generalizes beyond vision and can be applied to any set of models that process a common set of inputs, offering an approach for understanding what is shared and what is specific across learned representations.

## 6 Limitations

Several limitations and open questions remain. Our model set is broad but biased toward systems suited to tasks that humans find useful, and may not characterize the full space of possible visual representations. The additive, non-negative decomposition also cannot recover features that encode concepts at multiple levels of abstraction [24] or are stored in superposition [39, 41], and other methods may reveal additional structure in the model-specific components. Additionally, we used penultimate-layer representations because they were closest to behavior, but representations from other layers might modulate universality differently. Whether universal dimensions can be used to improve vision models, for instance, through representation alignment [51] or universality-based regularization during training, is another open question worth pursuing.

## Supplementary Material

Supplement 1
