# Supplementary material for: Characterizing Universal Object Representations Across Vision Models
Source: ArXiv. 2026 May 13:arXiv:2605.13675v1. Preprint. [Version 1] (PMC13317642)
Supplement: Supplement 1 [file NIHPP2605.13675v1-supplement-1.pdf]

A Model Overview

While no finite benchmark can represent all conceivable vision models, our set of 162 models span major contemporary sources of variation in visual representation, including architecture, objective, scale, training set and training task. Together with the robustness analyses showing stable universality rankings under model subsampling, exclusion of whole architecture families, and changes in image set (See Appendix C.5), this suggests that the universality score captures a robust recurrence pattern rather than a fragile artifact of the particular benchmark composition.

Table S1: **Overview of all 162 vision models.** Models are grouped by architecture class and sorted alphabetically within each group.

Model	Class	Family	Objective	Task	Data
2D Keypoints	CNN	ResNet	Supervised	Taskonomy	Taskonomy
3D Keypoints	CNN	ResNet	Supervised	Taskonomy	Taskonomy
Abstraction	CNN	ResNet	Supervised	BiT-Expert	BiT Transfer
AlexNet	CNN	AlexNet	Supervised	Supervised	ImageNet-1K
Animal	CNN	ResNet	Supervised	BiT-Expert	BiT Transfer
Arthropod	CNN	ResNet	Supervised	BiT-Expert	BiT Transfer
Autoencoder	CNN	ResNet	Supervised	Taskonomy	Taskonomy
Bird	CNN	ResNet	Supervised	BiT-Expert	BiT Transfer
CLIP-ResNet101	CNN	ResNet	Vision-Language	CLIP	OpenAI-400M
CLIP-ResNet50	CNN	ResNet	Vision-Language	CLIP	OpenAI-400M
CSP-ResNet50	CNN	ResNet	Supervised	Supervised	ImageNet-1K
Camera Pose (Fixated)	CNN	ResNet	Supervised	Taskonomy	Taskonomy
Camera Pose (Nonfixated)	CNN	ResNet	Supervised	Taskonomy	Taskonomy
ConvNext-B	CNN	ConvNeXt	Supervised	Supervised	ImageNet-1K
ConvNext-B-IN21K	CNN	ConvNeXt	Supervised	Supervised	ImageNet-21K
ConvNext-L	CNN	ConvNeXt	Supervised	Supervised	ImageNet-1K
ConvNext-L-IN21K	CNN	ConvNeXt	Supervised	Supervised	ImageNet-21K
Curvatures	CNN	ResNet	Supervised	Taskonomy	Taskonomy
DINO-ResNet50	CNN	ResNet	Self-Supervised	Self-Supervised	ImageNet-1K
DLA34	CNN	DLA	Supervised	Supervised	ImageNet-1K
Denoising	CNN	ResNet	Supervised	Taskonomy	Taskonomy
DenseNet121	CNN	DenseNet	Supervised	Supervised	ImageNet-1K
ECA-NFNet-L0	CNN	NFNet	Supervised	Supervised	ImageNet-1K
EfficientNet-B1	CNN	EfficientNet	Supervised	Supervised	ImageNet-1K
EfficientNet-B3	CNN	EfficientNet	Supervised	Supervised	ImageNet-1K
Egomotion	CNN	ResNet	Supervised	Taskonomy	Taskonomy
Euclidean Depth	CNN	ResNet	Supervised	Taskonomy	Taskonomy
Faster-RCNN-ResNet50-FPN	CNN	R-CNN	Supervised	Detection	COCO
Flower	CNN	ResNet	Supervised	BiT-Expert	BiT Transfer
Food	CNN	ResNet	Supervised	BiT-Expert	BiT Transfer
GMLP-S16	CNN	gMLP	Supervised	Supervised	ImageNet-1K
GMixer-24	CNN	gMixer	Supervised	Supervised	ImageNet-1K
GhostNet100	CNN	GhostNet	Supervised	Supervised	ImageNet-1K
GoogleNet	CNN	Inception	Supervised	Supervised	ImageNet-1K
HardCoreNAS-A	CNN	HardCoreNAS	Supervised	Supervised	ImageNet-1K
HardCoreNAS-F	CNN	HardCoreNAS	Supervised	Supervised	ImageNet-1K
Inception-V3	CNN	Inception	Supervised	Supervised	ImageNet-1K
Inpainting	CNN	ResNet	Supervised	Taskonomy	Taskonomy
Instrument	CNN	ResNet	Supervised	BiT-Expert	BiT Transfer
Jigsaw	CNN	ResNet	Supervised	Taskonomy	Taskonomy
Keypoint-RCNN-ResNet50-FPN	CNN	R-CNN	Supervised	Segmentation	COCO
MNASNet1.0	CNN	MNASNet	Supervised	Supervised	ImageNet-1K
Mammal	CNN	ResNet	Supervised	BiT-Expert	BiT Transfer
Mask-RCNN-ResNet50-FPN	CNN	R-CNN	Supervised	Segmentation	COCO
MiDaS	CNN	MiDaS	Supervised	Depth	Depth Mix
MobileNet-V2	CNN	MobileNet	Supervised	Supervised	ImageNet-1K
MobileNet-V3-Large	CNN	MobileNet	Supervised	Supervised	ImageNet-1K
NF-Net-L0	CNN	NFNet	Supervised	Supervised	ImageNet-1K
NF-ResNet50	CNN	ResNet	Supervised	Supervised	ImageNet-1K
Object	CNN	ResNet	Supervised	BiT-Expert	BiT Transfer
Object Classification	CNN	ResNet	Supervised	Taskonomy	Taskonomy

Model	Class	Family	Objective	Task	Data
Occlusion Edges	CNN	ResNet	Supervised	Taskonomy	Taskonomy
Point Matching	CNN	ResNet	Supervised	Taskonomy	Taskonomy
Random Weights	CNN	ResNet	Untrained	Untrained	Taskonomy
RegNet-128Gf-SEER	CNN	RegNet	Self-Supervised	SEER	Random-1B
RegNet-128Gf-SEER-INFT	CNN	RegNet	Self-Supervised	SEER	Random-1B
RegNet-32Gf-SEER	CNN	RegNet	Self-Supervised	SEER	Random-1B
RegNet-32Gf-SEER-INFT	CNN	RegNet	Self-Supervised	SEER	Random-1B
RegNet-64Gf-SEER	CNN	RegNet	Self-Supervised	SEER	Random-1B
RegNet-64Gf-SEER-INFT	CNN	RegNet	Self-Supervised	SEER	Random-1B
RegNetX-64	CNN	RegNet	Supervised	Supervised	ImageNet-1K
RegNetY-64	CNN	RegNet	Supervised	Supervised	ImageNet-1K
Relation	CNN	ResNet	Supervised	BiT-Expert	BiT Transfer
ResNet101	CNN	ResNet	Supervised	Supervised	ImageNet-1K
ResNet152	CNN	ResNet	Supervised	Supervised	ImageNet-1K
ResNet18	CNN	ResNet	Supervised	Supervised	ImageNet-1K
ResNet50	CNN	ResNet	Supervised	Supervised	ImageNet-1K
ResNet50-BarlowTwins	CNN	ResNet	Self-Supervised	Self-Supervised	ImageNet-1K
ResNet50-ClusterFit	CNN	ResNet	Self-Supervised	Self-Supervised	ImageNet-1K
ResNet50-DeepClusterV2	CNN	ResNet	Self-Supervised	Self-Supervised	ImageNet-1K
ResNet50-JigSaw-Goyal19	CNN	ResNet	Self-Supervised	Self-Supervised	ImageNet-1K
ResNet50-JigSaw-P100	CNN	ResNet	Self-Supervised	Self-Supervised	ImageNet-1K
ResNet50-MoCo-V2	CNN	ResNet	Self-Supervised	Self-Supervised	ImageNet-1K
ResNet50-PIRL	CNN	ResNet	Self-Supervised	Self-Supervised	ImageNet-1K
ResNet50-RotNet	CNN	ResNet	Self-Supervised	Self-Supervised	ImageNet-1K
ResNet50-SimCLR	CNN	ResNet	Self-Supervised	Self-Supervised	ImageNet-1K
ResNet50-SwAV	CNN	ResNet	Self-Supervised	Self-Supervised	ImageNet-1K
Reshading	CNN	ResNet	Supervised	Taskonomy	Taskonomy
RetinaNet-ResNet50-FPN	CNN	R-CNN	Supervised	Detection	COCO
Room Layout	CNN	ResNet	Supervised	Taskonomy	Taskonomy
SEResNext50-32x4D	CNN	SENet	Supervised	Supervised	ImageNet-1K
SKResNext50-32x4D	CNN	SENet	Supervised	Supervised	ImageNet-1K
Scene Classification	CNN	ResNet	Supervised	Taskonomy	Taskonomy
SemNASNet100	CNN	SemNASNet	Supervised	Supervised	ImageNet-1K
Semantic Segmentation	CNN	ResNet	Supervised	Taskonomy	Taskonomy
ShuffleNet-V2-x1.0	CNN	ShuffleNet	Supervised	Supervised	ImageNet-1K
SqueezeNet1.0	CNN	SqueezeNet	Supervised	Supervised	ImageNet-1K
Surface Normals	CNN	ResNet	Supervised	Taskonomy	Taskonomy
Texture Edges	CNN	ResNet	Supervised	Taskonomy	Taskonomy
Unsupervised 2.5D Segmentation	CNN	ResNet	Supervised	Taskonomy	Taskonomy
Unsupervised 2D Segmentation	CNN	ResNet	Supervised	Taskonomy	Taskonomy
VGG16	CNN	VGG	Supervised	Supervised	ImageNet-1K
Vanishing Point	CNN	ResNet	Supervised	Taskonomy	Taskonomy
Vehicle	CNN	ResNet	Supervised	BiT-Expert	BiT Transfer
Xception	CNN	Inception	Supervised	Supervised	ImageNet-1K
YOLO-V5-L	CNN	YOLO	Supervised	YOLO	COCO+VOC
YOLO-V5-M	CNN	YOLO	Supervised	YOLO	COCO+VOC
YOLO-V5-S	CNN	YOLO	Supervised	YOLO	COCO+VOC
Z-Buffer Depth	CNN	ResNet	Supervised	Taskonomy	Taskonomy
CLIP-ViT-B/16	ViT	ViT	Vision-Language	CLIP	OpenAI-400M
CLIP-ViT-B/32	ViT	ViT	Vision-Language	CLIP	OpenAI-400M
CLIP-ViT-L/14	ViT	ViT	Vision-Language	CLIP	OpenAI-400M
CoaT-Lite-Tiny	ViT	CoaT	Supervised	Supervised	ImageNet-1K
CrossViT-B	ViT	CrossViT	Supervised	Supervised	ImageNet-1K
DINO-ViT-B16	ViT	ViT	Self-Supervised	Self-Supervised	ImageNet-1K
DPT-Hybrid	ViT	DPT	Supervised	Depth	Depth Mix
DeiT-B-P16-224	ViT	DeiT	Supervised	Supervised	ImageNet-1K
JX-NesT-Tiny	ViT	NesT	Supervised	Supervised	ImageNet-1K
LeViT128	ViT	LeViT	Supervised	Supervised	ImageNet-1K
OpenCLIP-ViT-L/14 (DFN-2B)	ViT	ViT	Vision-Language	CLIP	DFN-2B
OpenCLIP-ViT-L/14 (DataComp-XL)	ViT	ViT	Vision-Language	CLIP	DataComp-XL
OpenCLIP-ViT-L/14 (LAION-2B)	ViT	ViT	Vision-Language	CLIP	LAION-2B

Model	Class	Family	Objective	Task	Data
OpenCLIP-ViT-L/14 (LAION-400M)	ViT	ViT	Vision-Language	CLIP	LAION-400M
OpenCLIP-ViT-L/14 (MetaCLIP-400M)	ViT	ViT	Vision-Language	CLIP	MetaCLIP-400M
OpenCLIP-ViT-L/14 (MetaCLIP-FullCC)	ViT	ViT	Vision-Language	CLIP	MetaCLIP-FullCC
PiT-B-224	ViT	PiT	Supervised	Supervised	ImageNet-1K
PiT-T-224	ViT	PiT	Supervised	Supervised	ImageNet-1K
PoolFormer-S36	ViT	PoolFormer	Supervised	Supervised	ImageNet-1K
Swin-B-P4-W7	ViT	Swin	Supervised	Supervised	ImageNet-1K
Swin-B-P4-W7-IN21K	ViT	Swin	Supervised	Supervised	ImageNet-21K
Swin-L-P4-W7	ViT	Swin	Supervised	Supervised	ImageNet-1K
Swin-L-P4-W7-IN21K	ViT	Swin	Supervised	Supervised	ImageNet-21K
Swin-T-P4-W7	ViT	Swin	Supervised	Supervised	ImageNet-1K
TnT-P16-224	ViT	TnT	Supervised	Supervised	ImageNet-1K
ViT-B-CLIP	ViT	ViT	Vision-Language	SLIP	YFCC-15M
ViT-B-P16	ViT	ViT	Supervised	Supervised	ImageNet-1K
ViT-B-P16-IN21K	ViT	ViT	Supervised	Supervised	ImageNet-21K
ViT-B-P32	ViT	ViT	Supervised	Supervised	ImageNet-1K
ViT-B-P32-IN21K	ViT	ViT	Supervised	Supervised	ImageNet-21K
ViT-B-R50-S16-IN21K	ViT	ViT	Supervised	Supervised	ImageNet-21K
ViT-B-SLIP	ViT	ViT	Vision-Language	SLIP	YFCC-15M
ViT-B-SimCLR	ViT	ViT	Self-Supervised	SLIP	YFCC-15M
ViT-L-CLIP	ViT	ViT	Vision-Language	SLIP	YFCC-15M
ViT-L-CLIP-CC12M	ViT	ViT	Vision-Language	SLIP	YFCC-15M
ViT-L-P16	ViT	ViT	Supervised	Supervised	ImageNet-1K
ViT-L-P16-IN21K	ViT	ViT	Supervised	Supervised	ImageNet-21K
ViT-L-SLIP	ViT	ViT	Vision-Language	SLIP	YFCC-15M
ViT-L-SLIP-CC12M	ViT	ViT	Vision-Language	SLIP	YFCC-15M
ViT-L-SimCLR	ViT	ViT	Self-Supervised	SLIP	YFCC-15M
ViT-S-CLIP	ViT	ViT	Vision-Language	SLIP	YFCC-15M
ViT-S-P16	ViT	ViT	Supervised	Supervised	ImageNet-1K
ViT-S-P16-IN21K	ViT	ViT	Supervised	Supervised	ImageNet-21K
ViT-S-P32	ViT	ViT	Supervised	Supervised	ImageNet-1K
ViT-S-P32-IN21K	ViT	ViT	Supervised	Supervised	ImageNet-21K
ViT-S-SLIP	ViT	ViT	Vision-Language	SLIP	YFCC-15M
ViT-S-SimCLR	ViT	ViT	Self-Supervised	SLIP	YFCC-15M
ViT-T-P16	ViT	ViT	Supervised	Supervised	ImageNet-1K
Visformer	ViT	Visformer	Supervised	Supervised	ImageNet-1K
XCIT-N-12-P16	ViT	XCiT	Supervised	Supervised	ImageNet-1K
XCIT-N-12-P8	ViT	XCiT	Supervised	Supervised	ImageNet-1K
MLP-Mixer-B16	Mixer	MLP-Mixer	Supervised	Supervised	ImageNet-1K
MLP-Mixer-B16-IN21K	Mixer	MLP-Mixer	Supervised	Supervised	ImageNet-21K
MLP-Mixer-L16	Mixer	MLP-Mixer	Supervised	Supervised	ImageNet-1K
MLP-Mixer-L16-IN21K	Mixer	MLP-Mixer	Supervised	Supervised	ImageNet-21K
ResMLP-12	Mixer	MLP-Mixer	Supervised	Supervised	ImageNet-1K
ResMLP-24	Mixer	MLP-Mixer	Supervised	Supervised	ImageNet-1K
ResMLP-36	Mixer	MLP-Mixer	Supervised	Supervised	ImageNet-1K
ResMLP-Big-24	Mixer	MLP-Mixer	Supervised	Supervised	ImageNet-1K
ResMLP-Big-24-IN21K	Mixer	MLP-Mixer	Supervised	Supervised	ImageNet-21K
ConViT-B	Hybrid	ConViT	Supervised	Supervised	ImageNet-1K
ConViT-T	Hybrid	ConViT	Supervised	Supervised	ImageNet-1K
ConvMixer-768-32	Hybrid	ConvMixer	Supervised	Supervised	ImageNet-1K

B Embedding generation

B.1 RBF Kernel and Bandwidth Selection

We define the symmetric similarity matrix $\mathbf{S}_m \in \mathbb{R}_{\geq 0}^{n \times n}$ via a radial basis function (RBF) kernel,

$$[\mathbf{S}_m]_{ij} = \exp\Big(-\frac{\|\boldsymbol{z}_{m,i} - \boldsymbol{z}_{m,j}\|^2}{2\sigma_m^2}\Big), \tag{6}$$

where the RBF kernel guarantees that $\mathbf{S}_m$ is positive semi-definite and nonnegative, both properties required by the symmetric nonnegative factorization (Eq. 2).

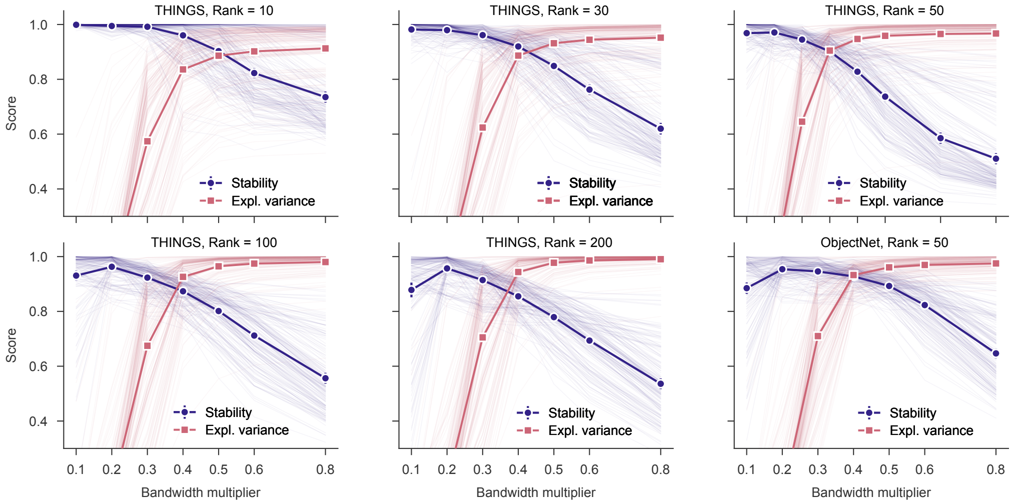

Figure S1: **Bandwidth selection.** Factorization stability and explained variance as a function of the RBF bandwidth multiplier α, for THINGS ranks 10, 30, 50, 100, and 200, and ObjectNet rank 50. Thin lines show individual models ($n = 162$); thick lines show means $\pm$ 95% CI. Dashed vertical lines indicate the optimal α^* (maximizing the harmonic mean of both criteria). The optimum is consistently near $\alpha = 0.4$–0.5 across ranks.

The bandwidth σ_m controls the emphasis on local versus global similarity structure. Rather than fixing it to the median pairwise distance, we set $\sigma_m = \alpha^* \cdot \tilde{d}_m$, where $\tilde{d}_m$ is the median pairwise Euclidean distance within model m [45] and α^* is chosen per model to jointly maximize factorization stability and explained variance. Specifically, we search over a grid of multipliers $\alpha \in \{0.1, 0.2, 0.3, 0.4, 0.5, 0.6, 0.8, 1.0\}$, run $B = 5$ random initializations per multiplier, and select the α^* that maximizes the harmonic mean of factorization stability (mean pairwise correlation of aligned solutions across seeds) and explained variance of the low-rank reconstruction, thus penalizing imbalanced solutions in which one criterion is optimal because another is sacrificed. Fig. S1 shows that factorization stability decreases monotonically with α while explained variance increases, producing a consistent optimum near $\alpha^* = 0.4$–0.5 across all ranks.

Note that we have

$$\frac{\partial [\mathbf{S}_m]_{ij}}{\partial \alpha} = \frac{\partial}{\partial \alpha} \exp\left(-\frac{\|\boldsymbol{z}_{m,i} - \boldsymbol{z}_{m,j}\|^2}{2\alpha^2 \tilde{d}_m^2}\right) = [\mathbf{S}_m]_{ij} \cdot \frac{\|\boldsymbol{z}_{m,i} - \boldsymbol{z}_{m,j}\|^2}{\alpha^3 \tilde{d}_m^2} > 0 \tag{7}$$

which implies when α increases, every off-diagonal similarity increases monotonically toward 1. More specifically, we can rewrite $\mathbf{S}_m$ with Taylor expansion around large α as

$$\mathbf{S}_m = \mathbf{1}\mathbf{1}^\top - \frac{1}{2\alpha^2 \tilde{d}_m^2}\mathbf{D} + O(\alpha^{-4}) \tag{8}$$

where $[\mathbf{D}]_{ij} = \|\boldsymbol{z}_{m,i} - \boldsymbol{z}_{m,j}\|^2$ is the squared pairwise Euclidean distance matrix. When α increases, less variance remains in higher-order directions, and $\mathbf{S}_m$ converges towards the leading term, which is rank 1. Consequently, for any fixed factorization rank r, the rank-r symmetric NMF can capture a larger fraction of similarity structure, leading to higher explained variance. At the same time, $\mathbf{S}_m$ contains less sharply differentiated structure as many pairs become similarly close. The matrix is dominated by broad global similarity. The optimization landscape for symmetric NMF becomes flatter in directions corresponding to splitting or merging of factors. The $WW^\top$ decomposition is then weakly constrained and therefore stability across runs drops.

B.2 Optimization

We optimize Eq. 2 via block successive upper-bound minimization [47]. For each model m, rank r, and candidate bandwidth multiplier, we run $B = 5$ random initializations. For each multiplier, we align solutions across initializations via the Hungarian algorithm and compute factorization stability

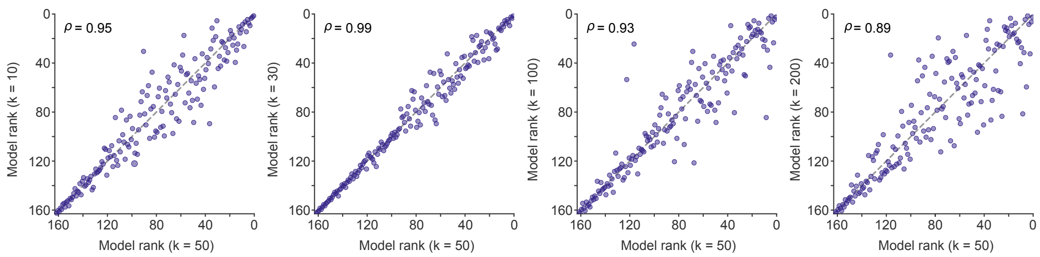

Figure S2: **Rank stability.** Model-level universality rankings at rank 50 compared against ranks 10, 30, 100, and 200. Each dot is one model; Spearman correlations are shown per panel.

as the mean matched correlation across seed pairs. After selecting the bandwidth multiplier by the harmonic mean of factorization stability and explained variance (Appendix B.1), we retain the most central seed, defined as the solution with the highest average matched correlation to all other seeds.

B.3 Rank Selection

We report results at rank $r = 50$ throughout the main text but repeat all analyses at $r \in \{10, 30, 100, 200\}$. Model-level universality rankings are highly stable across ranks (Fig. S2), with Spearman correlations between rank-50 and all other ranks at least $\rho = 0.93$ except for $r = 200$. This confirms that our conclusions do not depend on the particular factorization rank.

C Metric validation

C.1 Universality Metric Details

Our universality metric is used to test factor identity: whether an individual dimension recovered from one model recurs as an individual dimension in another model. This means that matching should be one-to-one, and scores should be calibrated against chance stimulus-level correspondence.

Why not greedy matching? A natural baseline assigns each target dimension its single best-matching source dimension. This greedy strategy permits many-to-one collisions: multiple target dimensions can claim the same source dimension, leaving others unmatched. Across all 26,082 model pairs at rank 50, 41% of source dimensions are never selected as any target dimension's best match (Fig. S3, a). Models with generic, broadly correlated dimensions benefit disproportionately from this inflation, while models with more distinctive dimensions are penalized. Because symmetric NMF factors are identifiable only up to permutation, a one-to-one assignment via the Hungarian algorithm is more principled: it respects the permutation structure of the factorization and ensures that each dimension receives exactly one match.

Null calibration. At moderate ranks, even unrelated dimensions can achieve non-trivial $\cos^2$ scores by chance. To remove this floor, we construct a permutation null for each target model m: we randomly permute the rows of source embeddings $\mathbf{W}_{m'}$, destroying stimulus correspondence while preserving column structure, recompute the Hungarian assignment and $\cos^2$-permutation scores on each shuffled dataset, and take the 95th percentile across $B = 1{,}000$ permutations as a per-dimension threshold $a_{m,k}$. The null-adjusted score is

$$s_{\text{adj}}(m, k;\, m') = \frac{s_{\pi^*}(m, k;\, m') - a_{m,k}}{1 - a_{m,k}}, \quad \text{clipped to } [0, 1], \tag{9}$$

and the final universality score averages these adjusted values across all remaining models. This calibration shifts the score distribution leftward and removes the positive floor present in the raw $\cos^2$-permutation scores (Fig. S3, b), ensuring that only dimensions with stimulus-specific correspondence contribute to universality.

Factor identity versus shared subspaces. The permutation-based universality score tests whether each factor in model m has a unique counterpart in model m'. Alternatively, one can ask whether

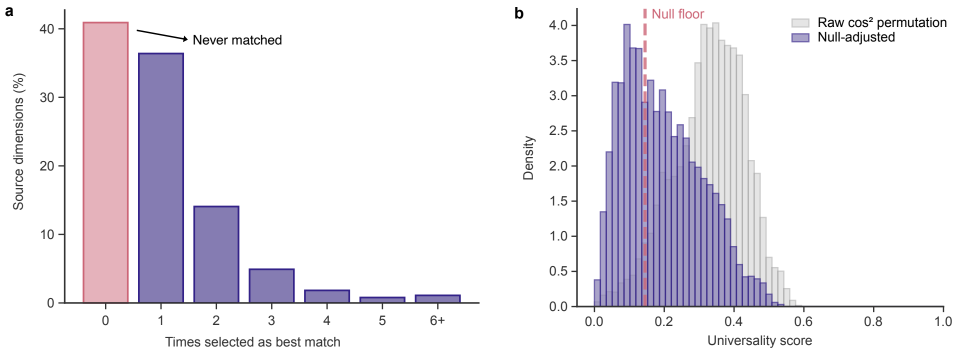

Figure S3: **Universality metric calibration.** **(a)** Distribution of how often each source dimension is selected as a target dimension's best match across all model pairs at rank 50. Under greedy matching, 41% of source dimensions are never selected, while others are claimed by multiple target dimensions. The Hungarian algorithm enforces exactly one match per dimension. **(b)** Distribution of per-dimension universality scores before (gray) and after (blue) null correction. Raw $\cos^2$-permutation scores exhibit a positive floor from chance alignment (dashed line); null adjustment removes this floor and rescales scores to $[0, 1]$.

a factor lies in the nonnegative cone of another model's factors, by projecting $\mathbf{w}_{m,k}$ onto the cone $\mathcal{C}(\mathbf{W}_{m'}) = \{\mathbf{W}_{m'}\mathbf{a} : \mathbf{a} \geq 0\}$ via nonnegative least squares and measuring how much variance is captured. This cone projection variant captures shared representational subspaces rather than strict factor identity, including cases where one model splits or merges concepts across dimensions. However, while this can detect broader shared structure, it also mixes level of description: the source is an individual factor, while the target is a model-level subspace (or vice-versa). Consequently, a high cone score is ambiguous, as it may reflect a true one-to-one counterpart, a split/merge correspondence across multiple factors, or only a diffuse approximation by several partially related factors. We use permutation matching as our primary metric because it directly tests factor identity, that is, whether the same dimension recurs across models.

C.2 Within-Model Stability Ceiling

To interpret universality on an absolute scale, we compute the within-model stability ceiling, defined as the typical agreement between independent NMF factorizations of the same model. Using the $B = 5$ seeds fit per model (Appendix B.2), we compute the $\cos^2$-permutation score between each of the $\binom{5}{2} = 10$ seed pairs, average across pairs per dimension, and apply the same null calibration as for universality (Appendix C.1).

The null-adjusted within-model stability has median 0.84 (IQR [0.75, 0.90], range [0.23, 0.995]) across all 8,100 dimensions. This is an empirical upper bound for cross-model agreement under $\cos^2$-perm, since a dimension cannot be more consistent across different models than it is across independent fits of a single model. Note that this ceiling varies only the NMF initialization while holding the underlying model fixed. A stricter ceiling that also varied model training, for example across independent training reruns of the same architecture, would likely be lower, making our estimate a liberal upper bound on cross-model agreement.

C.3 Universality Metric Validation

We summarize universality at the model level as $U_m = \frac{1}{r}\sum_{k=1}^{r} u_{m,k}$ and validate this metric in three ways (Fig. S4). First, we test whether universality generalizes to a different image set by recomputing scores from ObjectNet [48], which depicts objects in cluttered real-world scenes; model rankings are largely preserved. Second, we show that the $\cos^2$-permutation metric agrees closely with a cross-validated ridge regression variant at the per-dimension level. Third, we confirm that universality correlates strongly with centered kernel alignment (CKA), establishing convergent validity across different representational similarity measures.

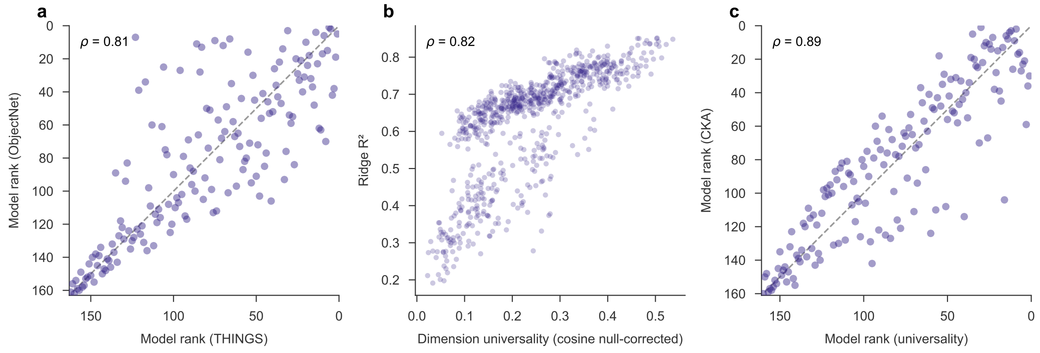

Figure S4: **Metric validation.** **(a)** Model-level universality rankings computed from THINGS vs. ObjectNet features ($\rho = 0.81$). **(b)** Per-dimension universality (null-corrected cosine) vs. cross-validated ridge R^2 ($\rho = 0.82$), confirming that our metric agrees with a more expensive regression-based proxy. **(c)** Model-level universality rank vs. CKA rank ($\rho = 0.89$), establishing convergent validity with a standard representational similarity measure.

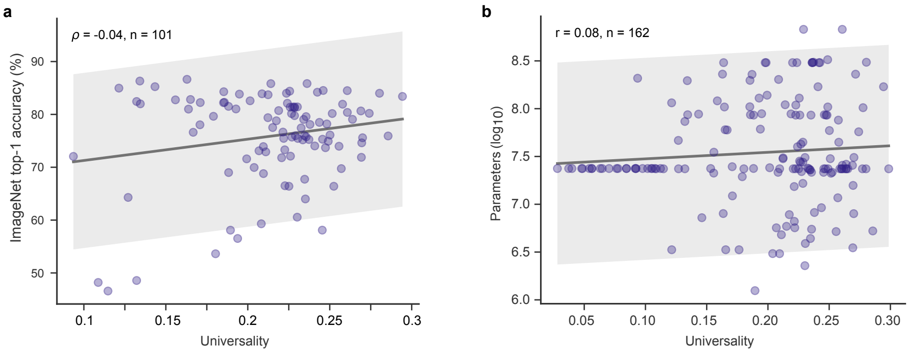

Figure S5: **Universality is not explained by model accuracy or size.** **(a)** Universality vs. ImageNet top-1 accuracy ($n = 101$; Spearman $\rho = -0.04$, $p = 0.72$). **(b)** Universality vs. number of parameters on a log scale ($n = 162$; Pearson $r = 0.08$, $p = 0.31$). Neither variable shows a reliable association with universality.

C.4 Universality vs. Model Accuracy and Size

Universality could in principle reflect model quality rather than shared representational structure. To rule this out, we correlate per-model universality with ImageNet top-1 accuracy (for the 101 models with reported accuracy) and with the total number of parameters (all 162 models). Neither shows a reliable association (Fig. S5). The Pearson correlation with accuracy is weak and non-significant ($r = 0.19$, $p = 0.052$), and the rank correlation is essentially zero ($\rho = -0.04$, $p = 0.72$), indicating that the marginal Pearson value is driven by a small number of outliers. The Pearson correlation with parameter count is similarly negligible ($r = 0.08$, $p = 0.31$). Models that are more accurate or larger are not systematically more universal.

C.5 Model-Set Stability

We test whether the universality metric is overly dependent on the specific model set over which it is computed.

Subsampling stability. We subsample the model set to 20% of models ($n = 32$, 1,000 iterations) and recompute universality scores. Model rankings based on overall universality are stable across iterations, with a median Spearman correlation between the full-set and subsampled rankings of $\rho = 0.97$ (95% range: $[0.84, 0.99]$).

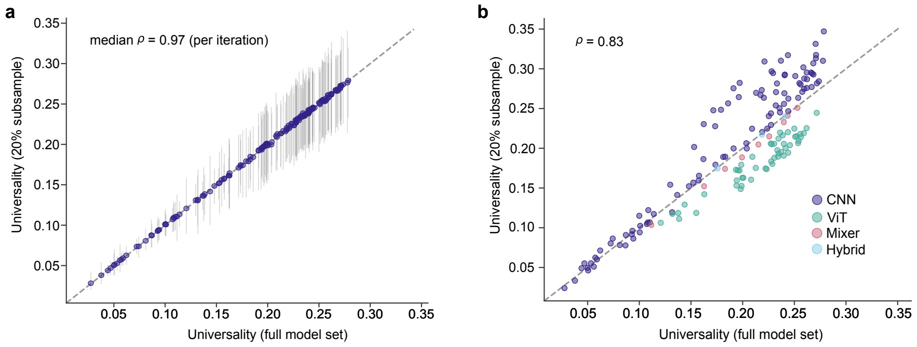

Figure S6: **Model-set stability.** **(a)** Bootstrap stability: universality from the full model set ($M = 162$) vs the mean across 1,000 bootstrap resamples (20% of models, $n = 32$). **(b)** Leave-family-out stability: universality from the full set vs recomputed after excluding all models from the same architecture family.

Leave-family-out stability. We also test whether the universality of dimensions in a given model depends on other models from the same architectural family being present in the set. Recomputing universality using only models from other architecture classes yields scores that are highly correlated with those of the full set ($\rho = 0.83$).

D Category Consistency

Our universality metric quantifies how consistently a dimension recurs across models, but does not reveal what a dimension represents. To characterize the content of each dimension, we exploit the categorical structure of the THINGS image set. The $N = 22{,}248$ images are organized into $C = 1{,}854$ object categories with $J = 12$ exemplars each ($N = C \times J$). Each NMF dimension $\boldsymbol{w}_{m,k} \in \mathbb{R}^N_{\geq 0}$ assigns a nonnegative loading to every image. If a dimension captures a semantic category, exemplars of the same category should receive similar loadings. If it instead reflects a visual property such as texture or color, exemplars within a category may receive very different loadings. We formalize this intuition using one-way ANOVA to decompose each dimension's loading variance into between- and within-category components.

For a given dimension k of model m, let $w_{m,k,cj}$ denote the loading of the j-th exemplar in category c, let $\bar{w}_{m,k,c} = \frac{1}{J}\sum_{j=1}^{J} w_{m,k,cj}$ be the mean loading for category c, and let $\bar{w}_{m,k} = \frac{1}{N}\sum_{c,j} w_{m,k,cj}$ be the grand mean over all images. The total sum of squares decomposes as

$$\underbrace{\sum_{c=1}^{C}\sum_{j=1}^{J}(w_{m,k,cj} - \bar{w}_{m,k})^2}_{\mathrm{SS}_{\mathrm{total}}} = \underbrace{\sum_{c=1}^{C} J\,(\bar{w}_{m,k,c} - \bar{w}_{m,k})^2}_{\mathrm{SS}_{\mathrm{between}}} + \underbrace{\sum_{c=1}^{C}\sum_{j=1}^{J}(w_{m,k,cj} - \bar{w}_{m,k,c})^2}_{\mathrm{SS}_{\mathrm{within}}}. \tag{10}$$

Category consistency is the proportion of variance explained by category membership,

$$\eta^2_{m,k} = \frac{\mathrm{SS}_{\mathrm{between}}}{\mathrm{SS}_{\mathrm{total}}} \in [0, 1]. \tag{11}$$

A dimension with $\eta^2_{m,k} \approx 1$ assigns nearly identical loadings to all exemplars of the same category and different loadings across categories. A dimension with $\eta^2_{m,k}$ near the chance level of $C/N \approx 0.08$ carries no more category information than expected from a random loading vector.

We compute $\eta^2_{m,k}$ independently for each dimension k in each model m, yielding r scores per model. To quantify reconstruction importance, we measure the drop in explained variance $\Delta R^2_{m,k}$ when dimension k is removed from the low-rank reconstruction $\mathbf{W}_m \mathbf{W}_m^\top$.

E Neural Predictivity

We use multi-unit activity (MUA) recorded from inferior temporal (IT) cortex of two rhesus macaques (F and N) viewing the same 22,248 THINGS object images [52]. Responses were z-scored per channel per recording day and averaged across the response time window by the original authors. Split-half reliability was pre-computed from a set of 100 test images presented with $\sim$30 repetitions each. We average reliability across splits to obtain a single reliability estimate per channel and retain only IT channels with mean reliability > 0.3, yielding 157 neurons for monkey F (of 320 IT channels) and 141 neurons for monkey N (of 256 IT channels).

For each of the 162 models, we fit a cross-validated ridge regression from the $r = 50$ NMF dimensions ($\mathbf{W}_m \in \mathbb{R}^{N \times r}$) to the response of each neuron independently. We use 5-fold cross-validation with ridge regularization selected within each training fold from $\alpha \in \{10^{-2}, \ldots, 10^{6}\}$ (20 log-spaced values). Encoding performance is the Pearson correlation between predicted and observed responses on held-out images, averaged across neurons and then across both monkeys to obtain a single encoding score per model. For the universal/specific-half comparison, we zeroed out the complementary 25 dimensions and reran the same cross-validated ridge procedure for each masked embedding.

We estimate a per-neuron noise ceiling as $\sqrt{\mathrm{reliab}_j}$, where reliab_j is the split-half reliability of neuron j [53]. Median noise ceilings are 0.71 (monkey F) and 0.83 (monkey N).

F Behavioral Predictivity

Dataset. We use the THINGS odd-one-out triplet judgments of Hebart et al. [13], collected on the 1,854 THINGS object categories. On each trial, a participant is shown three object images and chooses the one least similar to the other two (the odd-one-out). We use the public train and validation splits, yielding $4{,}120{,}663$ train and $457{,}430$ validation triplets ($4{,}578{,}093$ total). For the zero-shot analyses reported in the main text we pool train and validation triplets into a single evaluation set, since no parameters are fit to the triplets.

Image selection. For each of the $1{,}854$ categories we use the single image on which the human behavioral ratings of Hebart et al. [13] were collected, yielding a category-level embedding $\mathbf{W}_m^{\mathrm{cat}} \in \mathbb{R}_{\geq 0}^{1854 \times r}$ with $r = 50$.

Triplet accuracy. Let $\mathcal{T}$ denote the set of triplets, where each triplet is indexed by the triple of images (i, j, k) for which the participant selected k as the odd one out, so that (i, j) is the human-chosen similar pair. For model m, let $s_{ab}^m = \cos(\boldsymbol{w}_a^m, \boldsymbol{w}_b^m)$ denote the cosine similarity between rows a and b of the category-level embedding $\mathbf{W}_m^{\mathrm{cat}}$. The model's predicted similar pair is

$$(a^\star, b^\star) \;=\; \underset{(a,b) \in \{(i,j),\,(i,k),\,(j,k)\}}{\arg\max} \; s_{ab}^m, \tag{12}$$

and triplet accuracy is the fraction of triplets on which the predicted and human-chosen similar pairs agree

$$\mathrm{acc}(m) \;=\; \frac{1}{|\mathcal{T}|} \sum_{(i,j,k) \in \mathcal{T}} \mathbf{1}[(a^\star, b^\star) = (i, j)]\,. \tag{13}$$

Universal vs. specific half. To test whether universal dimensions drive behavioral alignment, we split each model's $r = 50$ dimensions at the median of the per-dimension universality score (Section 3.4) into a universal half (top 25 dimensions) and a model-specific half (bottom 25). For each half, we construct a masked embedding by zeroing out the complementary 25 columns of $\mathbf{W}_m^{\mathrm{cat}}$, recompute cosine similarities on the masked embedding, and evaluate triplet accuracy exactly as above. Significance is assessed with a paired t-test over the 162 models on the difference $\mathrm{acc}_{\text{universal}} - \mathrm{acc}_{\text{specific}}$.

G Compute Resources

All experiments were run on an internal high-performance computing cluster. Feature extraction from 162 models on THINGS and ObjectNet images required approximately 6 GPU-hours on NVIDIA

A100 40GB GPUs. Symmetric NMF across 162 models $\times$ 5 ranks $\times$ 5 random seeds (Eq. 2) required approximately 75,000 CPU-hours on CPU compute nodes with 512 GB RAM. Beyond the reported experiments, preliminary and exploratory runs required approximately an additional 5,000 CPU-hours.

H Human Dimension Rating Experiment

Goal. To characterize what each NMF dimension encodes from a human perspective, we collected crowd-sourced labels indicating whether each dimension reflects semantic content, visual properties, both, or neither.

Dimension selection. We applied hierarchical agglomerative clustering to all $162 \times 50 = 8{,}100$ dimensions using the precomputed linkage matrix (correlation distance). Re-cutting the dendrogram at a threshold of 0.75 yielded 1,059 clusters. For each cluster, we selected the single representative dimension whose weight vector $\boldsymbol{w}$ had highest correlation with the cluster centroid (mean weight vector), reducing redundancy while preserving the diversity of dimension types.

Stimuli. For each selected dimension, we constructed an image grid showing the 64 highest-loading images, with at most one image per THINGS category (to prevent any single category from dominating). Images were sorted in descending order of loading weight.

Task. Participants were shown image grids one at a time and asked to categorize each dimension by selecting one of four mutually exclusive labels:

- **Semantic** — the images share an identifiable object category or concept (e.g. all dogs, all vehicles).
- **Visual** — the images share a low-level perceptual property such as color, texture, or shape, without a clear shared concept.
- **Both** — the images share both a conceptual and a perceptual property.
- **Neither** — no clear shared property is apparent.

Participants also rated the difficulty of labeling each dimension on a 1–7 scale ($1 =$ very easy, $7 =$ very difficult).

Experimental design. The 1,059 dimensions were distributed across 71 survey versions using a between-subjects design, with 15 real trials per version. Each version additionally contained one catch trial (a grid of dog images, expected response: *semantic* or *both*), one attention-check trial (explicit instruction: select *both* and difficulty $= 4$), and one repeated real trial (the first real trial, re-shown at a random position, used to estimate within-participant consistency). Trial order within each version was randomized. Each version was assigned to 8 participants, initially yielding 8 assigned ratings per dimension before quality-control exclusions. The study was administered online via the Connect platform (see Fig. S7; participants were compensated at €0.75 per task (approximately 6 minutes per session or €7.50/h).

Quality control. Participants who failed the attention-check trial (i.e. did not select *both* with difficulty $= 4$) were excluded. Of 570 participants, 432 (76%) passed this criterion and were retained for analysis.

Reliability by content type. Overall, raters agreed with the majority-vote label on 65% of trials on average (per-rater mean accuracy = 0.65, median = 0.67), and majority-vote labels were stable across independent half-samples of raters, with the two halves agreeing on the majority label in 57% of splits on average (per-dimension median split-half reliability = 0.57, mean = 0.55). Both measures varied systematically across content categories. Per-rater accuracy was highest for *neither* dimensions ($\mu = 0.72$) and lowest for *both* dimensions ($\mu = 0.57$), with *semantic* and *visual* intermediate ($\mu \approx 0.63$; Kruskal–Wallis $H = 14.6$, $p = .002$). Split-half reliability followed the same ordering: *neither* dimensions yielded the most stable majority-vote labels (median = 0.77), while *both* dimensions were least stable (median = 0.35), with *visual* (median = 0.49) and *semantic* (median = 0.43) intermediate. This convergence across two independent reliability measures confirms

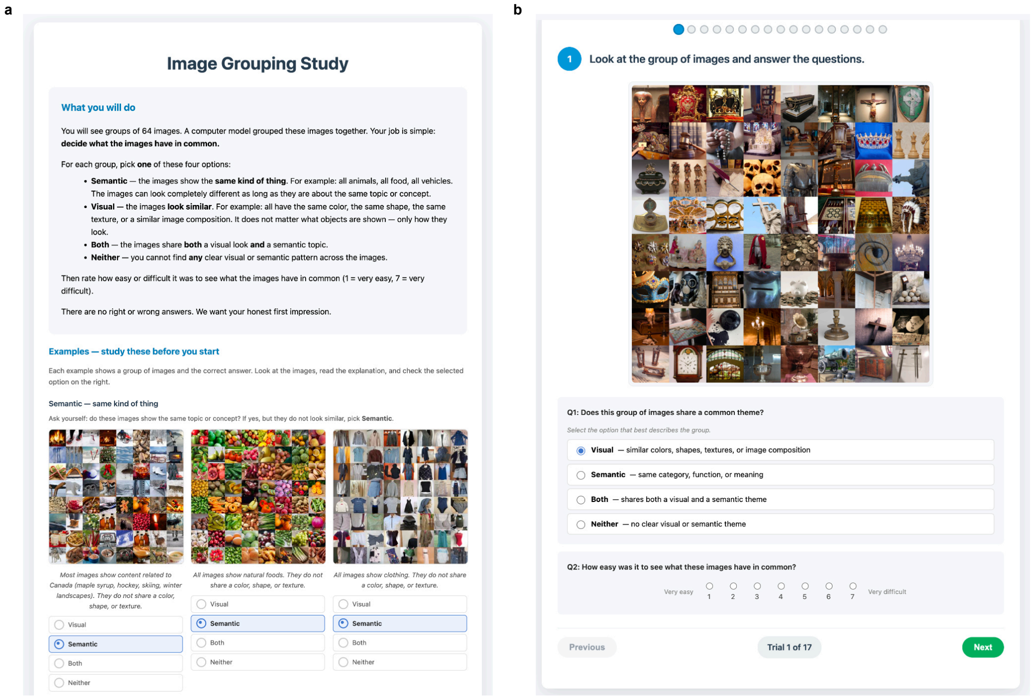

Figure S7: **Experiment instructions and task interface. (a)** Instructions provided to participants before the task, including visual examples of semantic, visual, mix and neither. **(b)** Task interface and questions asked.

that the observed pattern reflects genuine differences in label-boundary ambiguity rather than artifacts of any single metric. The structure of disagreements further validates the label space. When raters disagreed on a *both* dimension, they split nearly evenly between *semantic* (44%) and *visual* (42%), confirming that raters are decomposing a genuinely mixed-content dimension rather than responding randomly. When raters disagreed on a *visual* dimension, they most often substituted *neither* (43%), suggesting that the visual/neither boundary is the fuzziest in the label space – consistent with the observation that low-universality dimensions often encode subtle textural properties that can appear uninterpretable. Together, these patterns indicate that disagreements are structured and interpretable rather than random, supporting the validity of the majority-vote labels used throughout.
